# HerDing: herb recommendation system to treat diseases using genes and chemicals

**DOI:** 10.1093/database/baw011

**Published:** 2016-03-15

**Authors:** Wonjun Choi, Chan-Hun Choi, Young Ran Kim, Seon-Jong Kim, Chang-Su Na, Hyunju Lee

**Affiliations:** 1 School of Information and Communications, Gwangju Institute of Science and Technology, 123 Cheomdangwagi-ro, Buk-gu, Gwangju 61005, Republic of Korea; 2 College of Korean Medicine, Dongshin University, 185 Geonjae-ro, Naju-si, Jeollanam-do 58245, Republic of Korea and; 3 College of Pharmacy, Chonnam National University, 77 Yongbong-ro, Buk-gu, Gwangju 61186, Republic of Korea

## Abstract

In recent years, herbs have been researched for new drug candidates because they have a long empirical history of treating diseases and are relatively free from side effects. Studies to scientifically prove the medical efficacy of herbs for target diseases often spend a considerable amount of time and effort in choosing candidate herbs and in performing experiments to measure changes of marker genes when treating herbs. A computational approach to recommend herbs for treating diseases might be helpful to promote efficiency in the early stage of such studies. Although several databases related to traditional Chinese medicine have been already developed, there is no specialized Web tool yet recommending herbs to treat diseases based on disease-related genes. Therefore, we developed a novel search engine, HerDing, focused on retrieving candidate herb-related information with user search terms (a list of genes, a disease name, a chemical name or an herb name). HerDing was built by integrating public databases and by applying a text-mining method. The HerDing website is free and open to all users, and there is no login requirement.

**Database URL:** http://combio.gist.ac.kr/herding

## Introduction

**Figure 1.**
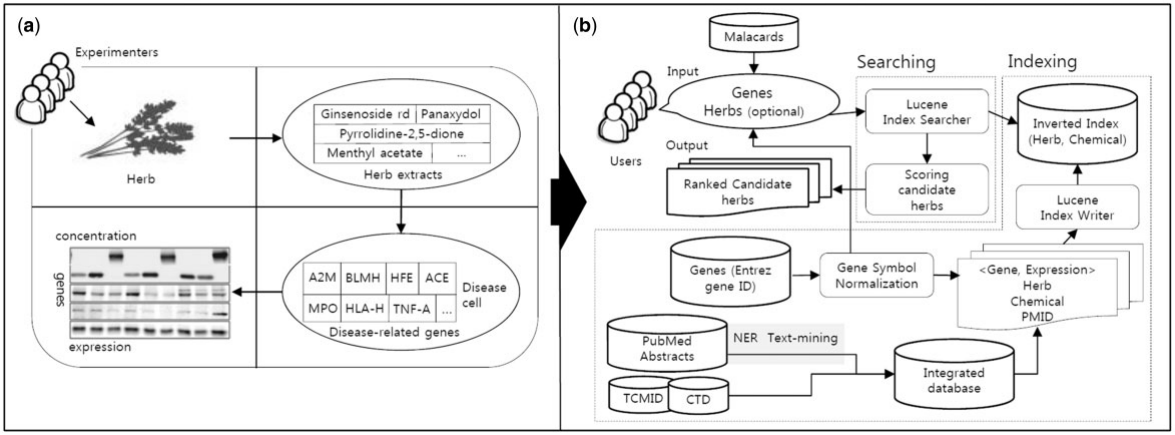
(**a**) An experiment to assess the effectiveness of herbs based on the changes of marker genes in a disease cell. (**b**) The process of the HerDing system consists of searching and indexing parts.

Natural products such as herbs have drawn increasing attention in finding new drug candidates in recent years because herbs have been practiced for treating diseases for thousands of years and might reduce side effects. With the increasing prevalence of chronic diseases such as cancer, diabetes, coronary heart disease and hypertension, natural product-based medicines are being popularized and have been used globally. Hence, many studies have been conducted to assess the effectiveness of natural products in treating diseases. For validation of the effects of herbs, experiments such as reverse transcriptase-polymerase chain reaction analysis and western blots have been widely used to measure the expression levels of disease-related genes or proteins in a disease cell (Figure 1a). For example, Wang *et al.* (1) assessed the therapeutic efficacy of the Realgar-Indigo naturalis formula in treating human acute promyelocytic leukemia (APL). For the assessment, the expression levels of marker genes of APL, including CD11b/CD14/CDK2, were measured in an APL-induced cell treated by the Realgar-Indigo naturalis formula.

Furthermore, studies for developing databases specializing in natural products have been actively conducted. Traditional Chinese Medicine Integrated Database (TCMID) (2) is one of the largest databases of oriental Chinese medicine, which records TCM-related information collected through a text-mining approach and by integrating different public databases including TCM-ID (3), HIT (4), TCM@Taiwan (5), STITCH (6), OMIM (7) and DrugBank (8). TCMID currently contains 8159 herbs, 25 210 compounds, 6828 drugs, 3721 diseases and 17 521 targets, and provides networks of relationships between various components such as herb-disease, herbal ingredients-targets and herbal-ingredient-target-disease-drug networks. TCM-ID (3) provides general TCM-related data including 3D structures of TCM compounds, which are manually collected from TCM-related books and other printed sources in Medline. TCM-ID currently contains 1313 herbs, 5669 herbal ingredients, 1588 prescriptions and the 3D structure of 3725 herbal ingredients. Also, TCM Database@Taiwan (5) is the world’s largest traditional Chinese medicine database specialized for *in silico* drug identification. It has collected >20 000 pure compounds isolated from 453 TCM herbs from Chinese medical texts and scientific publications.

In spite of the increasing interest in TCM-related databases, current Web applications contain a relatively small number of herbs compared to NCBI taxonomy (9), which contains >151 250 herbs in English name. This is partly because relationships between herbs and diseases were extracted only when they were directly mentioned in research articles. However, relationships between herbs and diseases can be expanded if disease-gene relationships are incorporated with herb information. In this study, we assume that if there is a resource that provides an association between marker genes of diseases and herbs, it would be helpful in recommending natural products for diseases. However, relationships between genes and herbs are not well understood. Hence, we used Swanson’s ABC model (10) to infer the influence of natural products on disease-related genes; a gene is targeted by a chemical, and a chemical is contained in a natural product. By bridging each relationship, we defined indirect relationships between herbs and genes (or diseases) and they were stored in our database. Then, we constructed a novel search engine, HerDing, which is a recommendation system to treat diseases based on the precomposed database and simulates the experimental process for measuring the efficacy of herbs in diseases (Figure 1a) with a computational approach (Figure 1b). The objective of HerDing is to assist researchers to reduce their time and experimental costs for proving herbs’ efficacy in treating diseases

## Materials and Methods

### Resources

HerDing integrated herb-chemical, chemical-gene and gene-disease relationships, which were obtained from other databases and text-mining of published articles, to find herb-disease relationships.

Among them, herb-chemical relationships were extracted by combining TCMID databases and a text-mining approach. We extracted herb-chemical relationships from TCMID, which provides 8159 herbs with 25 210 chemicals (as of March 2015). However, the number of herbs from TCMID is small compared to 151250 herbs in NCBI taxonomy. To enrich the relationships, we applied a rule-based text mining model to infer herb-chemical relationships (11). The text mining model consists of the following five rules: a verbal trigger rule, a prepositional trigger rule, a relative trigger rule, an apposition trigger rule and a copula trigger rule. An F-measure of the rule-based model was 0.749 when it was tested with 204 gold standard sentences. The rule-based model was applied to PubMed abstracts. For this task, herb names and chemical names were first identified from abstracts. For herb names, an herb name dictionary including English names, Chinese names and Latin names was constructed using TCMID and NCBI taxonomy, and then LingPipe (12), a dictionary-based exact matching NER tool, was used to locate herb names in abstracts. Chemical names were annotated using ChemSpot (13), which is a named entity recognition tool for locating chemical names including trivial names, drugs, abbreviations and molecular formulas in texts. When the rule-based model was applied to 13408 621 PubMed abstracts, a total of 101 550 herb-chemical relationships were extracted.

To construct the chemical-gene relationships, we integrated two public databases: The Comparative Toxicogenomics Database (CTD) (14) and TCMID. CTD provides detailed information about manually curated 1166896 chemical-gene interactions between 11 406 unique chemicals and 38433 genes in 540 organisms, 197288 chemical-disease relationships and 33814 gene-disease relationships. Because chemicals are presented using MeSH identifiers and CAS identifiers in CTD, whereas TCMID uses STITCH and PubChem (15) identifiers, we again used the ChemSpot NER tool to normalize chemical names. In addition, we normalized gene names to Entrez Gene identifiers using an identifier mapping tool provided by UniProt (16). Through this process, we accumulated 1240308 chemical-gene relationships from TCMID and CTD. Note that CTD provides directions of gene expression changes (i.e. up-regulated and down- regulated genes) in chemical-gene relationships.

Finally, we constructed herb-gene relationships by associating herb-chemical relationships and chemical-gene relationships using Swanson’s ABC model. Then, with the input of genes related to the disease, herb-disease relationships can be extracted. In summary, HerDing currently indexes 19 476 herb names, 16 762 gene names, 6655 chemical names and 11 394 diseases and provides 4880575 herb-gene relationships (as of November 2015).

### Indexing and searching

The indexing and searching process of the HerDing system is shown in Figure 1b. Indexing is the process of assigning a numerical value to a database entity to enable the quick search of queries in the search engine, and it is useful when there is a large number of entities. Lucene (17) is a high-performance text search engine library written in Java, and it provides a full text indexing and searching technology, supporting high access speed for multi-user accesses. Thus, we used Lucene to index database entities, which facilitated fast search results for users in HerDing. Through the indexing process, we constructed an inverted index file that indicates the location of relationships among herbs, chemicals and genes in the HerDing system. Thus, it allows rapid searches with input queries.

In the searching process, when the input is given by users, the Lucene index searcher accesses the inverted index file system to search relationships among herbs, chemicals and genes. In other words, herbs and chemicals with relationships with input genes will be collected by the Lucene index searcher. The scoring step is to measure the relevance of the candidate herbs, and the score of the herb was determined by the number of input genes related to a specific herb.

## Results

Users of HerDing can search herbs related to a disease with an input of disease-related genes or a disease name. In addition, users can query a chemical name for retrieving herbs and genes related to the input chemical and herb names for extracting chemicals and genes related to the input herb

### Example queries

The webpage of the HerDing system provides the following examples:

- *TNF, IL1B, IL6, IL8, IL17A, MMP1, MMP3, MMP9, MMP12, MMP13, COX2, IL10* *and IL4*: These genes are known as marker genes for rheumatoid arthritis. When they were input to HerDing, 30 (default) herbs were recommended. Among them, top-ranked herbs such as Radix Cudraniae (Chuan Po Shi) and *Polygonum cuspidatum* (Hu Zhang) were previously reported to be effective in treating rheumatoid arthritis (18–20).
- *Down-regulation (TNF, IL1B, IL6, IL8, IL17A, MMP1, MMP3, MMP9, MMP12, MMP13, COX2) and up-regulation (IL10, IL4)*: Down-regulation of the former set of genes and up-regulation of the latter set of genes are known to be a therapeutic strategy for rheumatoid arthritis. When they were input to HerDing, 30 (default) herbs were recommended. Among them, top-ranked herbs such as *Polygonum multiflorum* (He Shou Wu) and *Broussonetia papyrifera* (Gou Shu) were previously reported to reduce inflammation, which may be effective in treating rheumatoid arthritis (21, 22).
- *Hypertension*: When hypertension was given as an input from a user, 50 genes related to hypertension, which were extracted from MalaCards (23), were automatically replaced as the input to the HerDing system. As a result, 30 (default) herbs, including *Holothuria leucospilota* (Hai Shen), *Lycium chinense* (Gou Qi Zi), *Urtica dioica* (Yi Zhu Qian Ma) and soybean, were recommended. Among them, *H.* *leucospilota* and *L.* *chinense* were first- and third-ranked because chemicals contained in *H.* *leucospilota* and *L.* *chinense* target 22 and 18 genes out of the 50 input genes, respectively. Note that several studies (24–27) reported the effects of *H.* *leucospilota*, *L.* *chinense*, *U.* *dioica* and soybean on hypertension.
- *Saponins*: When a chemical name, saponins, was input to HerDing, 517 herbs that contain saponins and genes targeted by saponins were retrieved.
- *Dried tangerine peel*: When an herb, dried tangerine peel, was given as an input, 34 chemicals contained in the input herb and genes targeted by those chemicals were recommended.

### Web interface of the HerDing systems

We implemented HerDing’s web interface using the Apache Tomcat, an open-source software implementation of the Java Servlet and Java Server Pages (JSPs).

**Figure 2.**
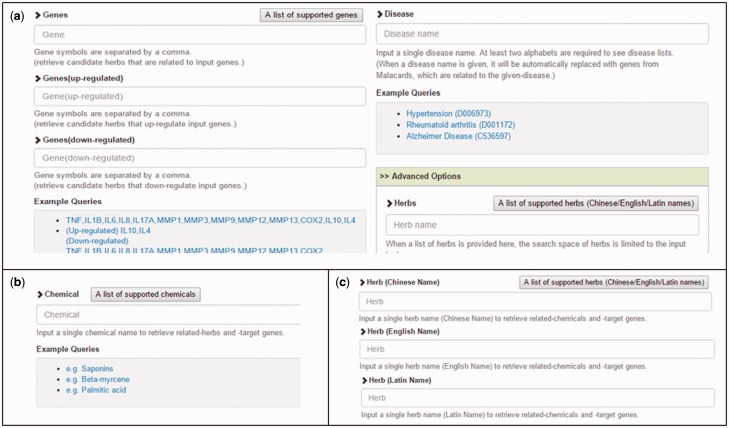
HerDing’s input pages. (**a**) Users can enter four different types of input in the each input box (a list of up-regulated genes, a list of down-regulated genes, genes regardless of the direction of expression changes, and a single disease name or a MeSH identifier). For advanced options, when users enter a list of herbs, the search space of herbs is limited to the input herbs. A list of supported herbs including Chinese/English/Latin names can be checked by clicking the ‘A list of supported herbs’ button. The number of maximum retrieved herbs can be changed. (**b**) Users can enter a chemical name to retrieve related herbs and target genes. (**c**) An herb name can be entered to retrieve chemicals containing the input herb and genes targeted by the chemicals.
- *Output*: Figure 3 shows a summary of results for a given user input (i.e. Alzheimer’s disease). The results page consists of three components including input information, a summary of retrieved herbs from input genes, and detailed information. From MalaCards, 50 genes related to Alzheimer’s disease are automatically retrieved, and they are used as inputs to HerDing. Then, the recommended herbs from HerDing are displayed in the order of the number of genes targeted by chemicals contained in the retrieved herbs. For example, in the second table of Figure 3, HAI SHEN is the top-ranked herb, which targets 25 Alzheimer-related genes. By clicking the ‘chemicals’ button, users can check a list of chemicals targeting the gene.

- ***Input***: Figure 2 shows the screen shots of web pages of HerDing and query interfaces for users, which contain three sections. In Figure 2a, HerDing’s query consists of disease-related genes. Genes can be specified in four different ways: (i) a list of up-regulated genes when an herb is treated; (ii) a list of down-regulated genes when an herb is treated; (iii) a list of genes (regardless of the direction of expression changes) and (iv) a single disease name or a MeSH identifier. (A list of genes will automatically be provided from the MalaCards (23) database when an input disease name or a MeSH ID is entered.) HerDing also provides two advanced options. When a list of herbs is provided as an input (optional), the search space of herbs is limited to the input herbs. The maximum number of herbs to be retrieved can be selected, on which the search speed depends. In Figure 2b, users can input a chemical name to retrieve herbs that contain the given chemical and genes targeted by the chemical. In Figure 2c, users can input an herb name to obtain chemicals that are contained in the given herb and genes targeted by the chemicals.

**Figure 3.**
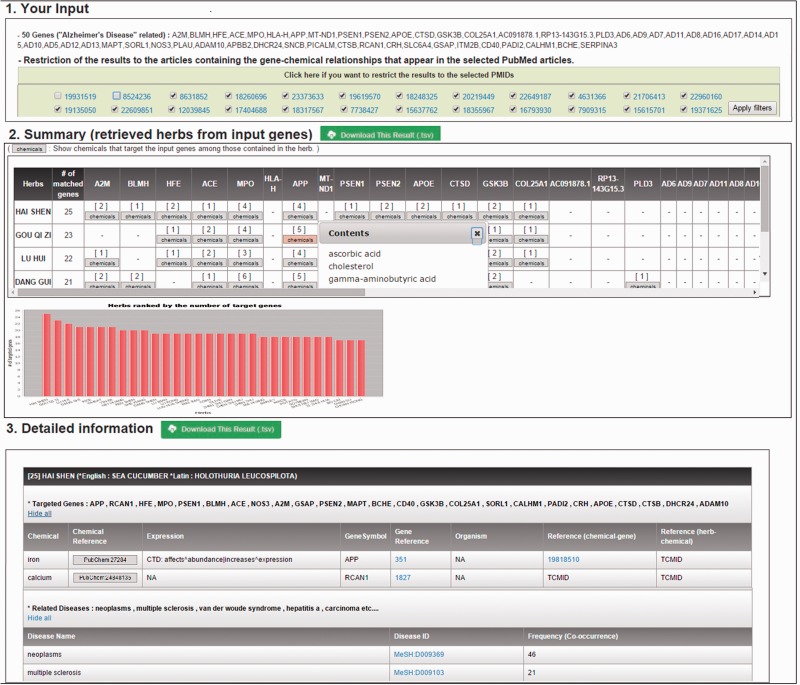
HerDing’s results page with an input of Alzheimer’s disease. HerDing displays user inputs, a summary of retrieved herbs, and detailed information. In the first section, users can check their input and restrict results to the selected articles. In the summary section, retrieved herbs with input genes are briefly presented in the table and the graph. Furthermore, chemicals targeting genes can be checked by clicking the ‘chemical’ button. In the detailed information section, users can obtain detailed information about herb-chemical-gene relationships and a list of diseases that are known to be related to a specific herb.

Furthermore, detailed information about the herbs is described in the third table of Figure 3. By clicking the ‘show all’ button, users can closely check detailed information such as active ingredients including the formula and 3D structure, the direction of expression changes of the targeted genes by chemicals, gene’ organisms and PMIDs that support chemical-gene relationships and herb-chemical relationships. A list of herb-related diseases is also provided. Herb-disease relationships in the detailed information were extracted based on the frequency of abstracts, where both an herb name and a disease name co-occurred in the same PubMed abstracts. Thus, highly ranked diseases would be the most related diseases that can be treated with a specific herb. Users can optionally restrict results of queries to gene-chemical relationships, which were contained in PubMed abstracts selected by clicking ‘apply filters’, as shown in the first table of Figure 3. This allows researchers to use HerDing as a search prioritization tool based on articles that are more appropriate for their research design. All the retrieved results are freely downloadable by clicking a button denoted as ‘download this result(.tsv)’ in the results page and stored in the tab-separated file.

When users input a chemical name or an herb name, the summary and detailed information in Figure 3 are changed. With a query of a chemical name, a list of herbs that contain the input chemical and a list of genes targeted by the chemical are shown. With a query of an herb name, a list of chemicals contained in the given herb and a list of genes targeted by the chemicals are presented.

### Evaluation of the recommended herbs

To evaluate the HerDing system, we examined 20 diseases including Alzheimer’s disease and diabetic neuropathies (Supplementary Table S1). Genes related to a given disease were collected from MalaCards. When we manually examined the top ten recommended herbs for each disease, research articles supporting the effectiveness of herbs for the disease were retrieved, as shown in Supplementary Table S1. For example, sea cucumber, wolfberry fruit and aloe vera were recommended for Alzheimer’s disease (ranked first, second and third, respectively), and their effectiveness has previously been reported. Similarly, it was reported that *Angelica* sinensis, *Curcuma* longa and *Portulaca oleracea* (ranked first, second and third, respectively) were effective for the treatment of diabetic neuropathies.

Jeoung *et al.* (28) reported that Ganghwaljetongyeum (GHJTY), a complex herbal formula consisting of 18 herbs, was effective for the treatment of rheumatoid arthritis. When we searched HerDing using 50 genes related to rheumatoid arthritis and 18 herbs from GHJTY, all 18 herbs were found to target at least one gene out of 50 genes. We also observed that *Chinese angelica* (Dang Gui) and *clematis chinensis* (Wei Ling Xian) were highly ranked herbs, targeting 21 and 10 genes, respectively, whereas both oriental water plantain (Ze Xie) and five leaf akebia (Mu Tong) target a single gene. This result implies that further research to discover a simpler herbal formula using a few top-ranked genes can be conducted in treating rheumatoid arthritis.

## Summary and Discussion

HerDing is a search engine for recommending herbs to treat diseases and was constructed based on the assumption that an association between marker genes of diseases and herbs would draw indirect relationships between herbs and diseases. These herb-chemical-gene-disease indirect relationships were extracted by integrating public resources and by using the text-mining approach. Although several Web-based databases in the field of traditional Chinese medicine, including TCMID (2), have been constructed, most of them provide herb-disease relationships when the relationships were directly mentioned in research articles. With the increasing knowledge about molecular mechanisms of diseases, diseases have been researched based on genes. The novelty of HerDing is that users can retrieve candidate herbs for treating diseases based on multiple disease-related genes, which allows users to explore more candidate herbs.

Although HerDing provides new insights in searching herb-disease relationships, there is still much room for improvement by providing more abundant and accurate data. To extract information from less-studied herbs, a high-performance text-mining method is required. Thus, if we update the dictionary with more herb names and develop an accurate NER tool, the number of herb-chemical relationships will be increased. In addition, developing the machine learning approaches for extracting herb-chemical relationships will be a future work. To increase the database size, other text resources related to natural products, such as news articles, electronic books, Wikipedia and Google, will be useful.
